# Deubiquitylating enzymes and disease

**DOI:** 10.1186/1471-2091-9-S1-S3

**Published:** 2008-10-21

**Authors:** Shweta Singhal, Matthew C Taylor, Rohan T Baker

**Affiliations:** 1 Ubiquitin Laboratory, Division of Molecular Bioscience, The John Curtin School of Medical Research, The Australian National University, Canberra, ACT 0200, Australia; 2 CSIRO Entomology, GPO Box 1700, Canberra, ACT 2601, Australia

## Abstract

Deubiquitylating enzymes (DUBs) can hydrolyze a peptide, amide, ester or thiolester bond at the C-terminus of UBIQ (ubiquitin), including the post-translationally formed branched peptide bonds in mono- or multi-ubiquitylated conjugates. DUBs thus have the potential to regulate any UBIQ-mediated cellular process, the two best characterized being proteolysis and protein trafficking. Mammals contain some 80–90 DUBs in five different subfamilies, only a handful of which have been characterized with respect to the proteins that they interact with and deubiquitylate. Several other DUBs have been implicated in various disease processes in which they are changed by mutation, have altered expression levels, and/or form part of regulatory complexes. Specific examples of DUB involvement in various diseases are presented. While no specific drugs targeting DUBs have yet been described, sufficient functional and structural information has accumulated in some cases to allow their rapid development.

Republished from Current BioData's Targeted Proteins database (TPdb; ).

## Localization and function

### Introduction

In this chapter, the term 'deubiquitylating enzyme' (DUB) is used to describe any enzyme that can hydrolyze a peptide, amide, ester or thiolester bond at the C-terminus of UBIQ (ubiquitin). DUBs can cleave the linear products of UBIQ gene translation [1]; the post-translationally formed branched peptide bonds in mono- or multi-ubiquitylated conjugates [2]; ubiquitylated remnants resulting from proteasome-mediated degradation [3] and other small amide or ester adducts [3,4]. DUBs thus have the potential to regulate any UBIQ-mediated cellular process, the two best characterized being proteolysis and protein trafficking/endocytosis.

In mammals there are some 80–90 DUBs categorized into five gene families: the ubiquitin C-terminal hydrolases (UCHs); the ubiquitin-specific peptidases (USPs/UBPs); the ovarian tumor (OTU) domain proteins; the Josephin or Machado-Joseph disease (MJD) proteins and the JAMM (Jab1/MPN domain-associated metalloisopeptidase) domain proteins. The first four families are cysteine peptidases, while the JAMM proteins are zinc metalloisopeptidases. These DUB families have been the subjects of recent reviews [5-7]. Since linear UBIQ fusion proteins are cleaved very rapidly, perhaps co-translationally [1], it is unclear which DUB(s) cleave these precursors *in vivo *to provide free UBIQ. However, mutating or inhibiting a DUB(s) that produces free UBIQ would have pleiotropic effects, in that it would deplete free UBIQ levels and inhibit all UBIQ-dependent functions non-specifically. DUBs that cleave branched UBIQ conjugates presumably make up the bulk of the 80–90 DUBs, and provide substrate specificity to deubiquitylation. Most DUBs contain a catalytic domain that has sequence similarity within subfamilies and structural similarity across subfamilies [5-8], and unrelated sequences either N-terminal or C-terminal (or both) to the catalytic domain. These flanking sequences have been shown to mediate substrate binding in a few cases (see *DUBs and disease: UBP7/USP7/HAUSP *and *DUBs and disease: UBP33/USP33/VDU1, UBP20USP20/VDU2*) and presumably serve as substrate binding domains in all DUBs. These flanking sequences, along with the catalytic core, could also contribute binding and cleavage specificity for different UBIQ-UBIQ linkages. Some DUBs function at the proteasome to edit and/or remove UBIQ chains; one example that is linked to disease is UBP14 (USP14) (see *DUBs and disease: UBP14/USP14*).

Since most DUBs have been identified only by means of sequence similarity to catalytic motifs, there is little known functional information on many of these enzymes. However, the relatively few examples where functional insights have been gained (see *DUBs and disease*) indicate that DUBs can play crucial regulatory roles in the ubiquitin proteasome system (UPS), making them ideal drug target candidates for therapeutic intervention in UPS-related diseases.

### DUBs and disease

#### UBP6/USP6/TRE-17/TRE-2

UBP6 (encoded by *USP6/TRE-17*), the first DUB to be identified as an oncogene [9,10], has in recent years been directly linked to human cancers, primarily aneurysmal bone cysts (ABCs), which are locally aggressive bone tumors. ABCs were previously regarded as non-neoplastic, but recent cytogenetic studies have identified clonal rearrangements that often feature chromosome 17p13 – the *USP6 *or *TRE-17 *(originally termed *TRE-2*) locus. There are five known examples of chromosomal rearrangements that have positioned *USP6 *downstream of a heterologous gene promoter, in turn forcing inappropriate *USP6 *expression in a bone/mesenchymal context: *Osteomodulin*; *COL1A1 (Collagen 1A1)*; *THRAP3 (TRAP150)*; *CNBP (ZNF9) *and *CDH11 *[11-13]. High-level UBP6 expression was also detected in four other human cancers originating from mesenchymal neoplastic cells in a bone context (one Ewing's sarcoma, two osteoblastomas and one myofibroma), but not in 50 other non-ABC tumors, suggesting that UBP6 could have a broader oncogenic role in mesenchymal tumors [13]. Recent work has also revealed that the *USP6 *product regulates actin remodeling and vesicular trafficking, and could thus regulate cell motility and invasiveness [14]. In all five cases of UBP6-linked human cancers referred to above, it remains unclear whether the heterologous promoters cause overexpression of normal, full-length UBP6 protein, or whether there have been further mutations, deletions, or alternate splicing within *USP6 *to produce an altered, oncogenic UBP6 protein.

#### UBP7/USP7/HAUSP

One well characterized case that illustrates the possible link between DUBs and disease is the mammalian DUB UBP7 (encoded by *USP7/HAUSP*), a 1102 amino acid member of the USP family. The N-terminal domain of UBP7 (residues 1–208) contains a TRAF (tumor necrosis factor receptor-associated factor) domain [15] that binds to both the P53 (p53) tumor suppressor protein [16,17] and the MDM2 ubiquitin ligase, which ubiquitylates P53 [18]. The remainder of UBP7 comprises a catalytic core (residues 208–560) that cleaves UBIQ, and a C-terminal domain (residues 560–1102) of unknown function. Since UBP7 can deubiquitylate and stabilize P53 *in vitro*, it was suggested that the role of UBP7 was to stabilize P53 *in vivo *[16]. More recent work, however, showed that UBP7 can also deubiquitylate and thus stabilize MDM2 (which autoubiquitylates itself) [19,20]. UBP7 forms a tighter complex with MDM2 than with P53 [21], consistent with observations that UBP7's primary role could be deubiquitylating and stabilizing MDM2 (and thus increasing ubiquitylation of P53), rather than deubiquitylating and stabilizing P53 [20]. MDM2 is not the only P53 ubiquitin ligase and additional proteins that play a role in P53 ubiquitylation, deubiquitylation and degradation have been recently reviewed [22]. One further member of this complex interplay was recently identified as the protein DAXX (death domain-associated protein). DAXX simultaneously binds to MDM2 and UBP7, and mediates the stabilizing effect of UBP7 on MDM2 [23]. In response to DNA damage, DAXX (and UBP7) dissociate from MDM2, which correlates with MDM2 self-degradation.

Pathogenic mutations within UBP7 itself have not yet been described. However, a recent study that investigated UBP7 expression and *TP53 *gene status in non-small cell lung carcinomas found that, in 93 of the 131 patients examined, either mutant P53 or reduced UBP7 expression was observed [24]. A statistically significant association between reduced UBP7 levels and reduced P53 protein expression was observed in tumors with wild-type P53, while a more dramatic association was seen in tumors with mutant P53. The authors concluded that the concurrent evaluation of both UBP7 expression and P53 gene status was a significant prognostic indicator in adenocarcinoma patients [24]. MDM2 expression was not investigated in this study though it could be another useful prognostic indicator, given that the protein can regulate P53 levels.

Several disease-causing viruses also target P53 levels by manipulating the interaction between UBP7 and P53. These include the HSV protein ICPO (which is a ubiquitin RING finger ligase that targets several cellular proteins for degradation, including P53 [25]), and the Epstein-Barr nuclear antigen 1 (EBNA1) protein, which displaces UBP7 from P53 [26].

In addition to having multiple targets in the same pathway (i.e. P53, MDM2 and MDM4 [MDMX]), UBP7 has at least one other non-proteolytic target. The transcription factor forkhead box O (FOXO) becomes monoubiquitylated in response to increased cellular oxidative stress, resulting in its re-localization to the nucleus and an increase in its transcriptional activity. UBP7 removes UBIQ from monoubiquitylated FOXO, and negatively regulates FOXO transcriptional activity towards endogenous promoters [27]. Thus, we must keep in mind that DUBs not only regulate protein degradation, but also protein trafficking/localization.

#### CYLD

Mutations in the cylindromatosis protein (CYLD), a tumor suppressor, are linked to familial cylindromatosis (MIM132700), an autosomal dominant predisposition to multiple neoplasms of skin appendages [28,29]. The C-terminal 365 amino acids of the 953-residue CYLD protein comprise a variant USP-type DUB catalytic core, and CYLD has been shown to possess deubiquitylating activity both *in vitro *and in whole cells. This deubiquitylating activity was specific to non-Lys48-linked UBIQ chains [30]. CYLD functions to downregulate NFκB signaling, in which the UPS has several roles [30]. Upon receptor activation, TRAF2, TRAF6 and the NEMO (IKKγ) subunit are polyubiquitylated with Lys63-linked UBIQ chains, which is a necessary process to activate the IKK complex to phosphorylate IκB. IκB in turn becomes polyubiquitylated with Lys48-linked ubiquitin chains and is degraded, releasing NFκB for translocation to the nucleus to enable gene activation [30-32]. CYLD acts to downregulate NFκB signaling by removing these activating Lys63-linked UBIQ chains [30]. Reduced or absent CYLD activity allows prolonged NFκB signaling, increases resistance to apoptosis and hence could lead to tumor formation [31]. CYLD is expressed in a broad range of tissue types, and it remains unclear as to why CYLD mutations only give rise to skin tumors [33].

In a study of 25 cylindromatosis families, 21 had CYLD mutations resulting in truncations or frameshift alterations within the C-terminal two-thirds of the protein that would abolish its deubiquitylating activity, thus indicating a correlation between tumorigenesis and reduced deubiquitylating activity [33]. Recent studies on CYLD-deficient mice demonstrated a deficiency in T-cell development [34], and observed that activation of their immune cells led to increased NFκB signaling, a higher susceptibility to induced colonic inflammation and increased incidence of tumors when compared with controls in a colitis-associated cancer model [35].

#### TNAP3/A20/TNFAIP3

Another DUB that plays a role in downregulating NFκB signaling is TNAP3 (A20, encoded by *TNFAIP*), which has an OTU DUB domain. Previous studies of yet another negative regulator of NFκB signaling, OTU7B (Cezanne), determined that the N-terminal OTU domain of this protein conferred its DUB activity [36], which was subsequently shown for TNAP3 [37]. TNA3P can cleave both Lys48- and Lys63-linked UBIQ chains *in vitro*, but *in vivo *appears to have specificity for Lys63 chains. However, TNAP3 also possesses a novel zinc finger-type ubiquitin ligase (E3) domain, which can assemble Lys48-linked UBIQ chains *in vitro *and in transfected cells [37]. Thus, TNAP3 acts as an inhibitor of NFκB signaling by removing the (activating) Lys63 chains on the tumor necrosis factor (TNF) receptor-interacting protein RIP, and then assembling Lys48 linked chains on RIP by virtue of its ubiquitin ligase domain. This results in the degradation of RIP, thus preventing its activation of NFκB via the TNF-mediated pathway [37].

To date, no TNAP3 mutations have been linked to human disease. However, TNAP3-deficient mice develop severe inflammation and cachexia, are hypersensitive to both lipopolysaccharide and TNF, and die prematurely, consistent with failure to terminate TNF-induced NFκB responses [38]. The role of the UBIQ pathway in NFκB signaling has been extensively reviewed [39-41].

#### UBP33/USP33/VDU1, UBP20/USP20/VDU2 and von Hippel-Lindau disease

Von Hippel-Lindau disease is an autosomal dominant disorder that predisposes affected individuals to a variety of tumors, including hemangioblastomas in the CNS and retina, clear cell carcinomas of the kidney, pheochromocytomas of the adrenal gland, and pancreatic cysts, adenomas and islet cell tumors (reviewed in [42]). UBP33 (encoded by *USP33/VDU1*) and UBP20 (encoded by *USP20/VDU2*) are 59% identical USP-type DUBs that interact with the tumor suppressor E3 ubiquitin ligase VHL (pVHL), mutations in which are associated with von Hippel-Lindau disease [43]. UBP33 and UBP20 interact with the β-domain of VHL, leading to their ubiquitylation and degradation by the proteasome [44]. The β-domain of VHL is the region of the protein that harbors the naturally occurring mutations found in von Hippel-Lindau disease. Some of these mutations have been shown to disrupt UBP33/20 interaction with VHL, suggesting an important role for UBP33/20 in this disease [43,44]. One target of VHL is the α-subunit of the transcription factor hypoxia-inducible factor-1 (HIF-1) [45], which regulates the genes involved in angiogenesis, glucose metabolism, cell proliferation, invasion and metastasis [46]. The inability to degrade HIF1A (HIF-1α) leads to overexpression of HIFA target genes and to a variety of tumors [42]. Recently, it has been shown that UBP20, but not UBP33, interacts with HIFIA and can specifically deubiquitylate and stabilize it, antagonizing VHL-mediated ubiquitylation of the transcription factor [45]. This UBP20/HIF1A/VHL interplay is functionally similar to the UBP7/P53/MDM2 situation described previously (see *DUBs and disease: UBP7/USP7/HAUSP*).

#### UBP14

UBP14 (encoded by *ubp14/gid6*) is a USP-type DUB that is localized to the proteasome by virtue of a ubiquitin-like domain N-terminal to its catalytic core. This DUB has been best studied in yeast, where it was found that UBP14 activity was increased 300-fold upon binding to the proteasome, and it was originally proposed to assist in the release of UBIQ from proteasome-bound multi-ubiquitylated conjugates [47]. In yeast, loss of UBP14 depletes free cellular UBIQ levels due to increased degradation of UBIQ by the proteasome, and renders cells susceptible to stresses that impose extra load on UBIQ-dependent proteolysis [47]. However, more recent studies suggest that UBP14 regulates proteasome activity by actually delaying UBIQ chain removal, and that it is involved in a dynamic remodeling of UBIQ chains at the proteasome in conjunction with a proteasome-bound ubiquitin ligase, HUL5 [48,49]. In mammals, UBP14 (encoded by *USP14*) is also associated with the proteasome, and loss of UBP14 in mice also leads to depletion of UBIQ [50], resulting in an ataxia phenotype due to defects in synaptic transmission [51]. However, it is not yet clear whether this is due to a general defect in proteolysis (by analogy to the yeast *ubp14 *deletion mutant) or to a more specific, perhaps non-proteasomal role for UBP14 in neurons.

#### UCHL1

Ubiquitin C-terminal hydrolase isozyme L1 (UCHL1) catalyzes the hydrolysis of C-terminal ubiquityl esters and amides, releasing UBIQ from substrates [4]. UCHL1 is a highly abundant neuronal enzyme, comprising up to 2% of total brain protein [52]. Although it has mainly been implicated in deubiquitylation, it has also been shown *in vitro *to have ubiquitin ligase activity and this activity has been correlated with dimerization of the enzyme. Mutations in the *UCHL1 *gene have been linked to susceptibility to and protection from Parkinson's disease (PD) [53,54]. UCHL1 was initially linked to PD in a German family [55] where a point mutation at nucleotide C_277_G led to the amino acid substitution Ile93Met [56,57]. The Met93 variant has a severely diminished hydrolase activity and a lower E3 ligase activity compared with wild-type UCHL1. UCHL1 is found in Lewy body protein aggregates associated with PD. These Lewy bodies amass a range of normal and abnormal proteins, many of which are ubiquitylated [58]. However, this polymorphism has only been observed in one PD family. The second polymorphism in UCHL1 results in an amino acid change, Ser18Tyr [59]. This mutation is, however, protective against PD [60] and delays the age of onset of disease. The Tyr18 allele prevents the formation of UCHL1 dimers and therefore lacks ubiquitin ligase activity. In addition, the Tyr18 allele has been shown to increase the hydrolase activity of UCHL1 *in vitro *[61,62]. Taken together, these results suggest that increased UCHL1 hydrolase activity is protective against PD, but that the specific substrates of UCHL1 require further investigation.

#### ATX3

Spinocerebellar ataxia (SCA) type-3 or Machado-Joseph disease (MJD) is a member of the CAG/polyglutamine repeats disease family [63,64]. Expanded polyglutamine confers a toxic gain of function on the disease protein, presumably through an increased propensity towards aggregation, altered protein expression or both [65]. ATX3 (Ataxin-3), the disease protein associated with SCA3/MJD, is a ubiquitously expressed protein and a member of a novel family of DUBs defined by the Josephin or MJD domain [66-68]. Normally, the protein contains 12–40 glutamines near its C-terminus, whereas in disease the polyglutamine domain expands to ~55–84 glutamines. Expression of mutant ATX3 is widespread, although the neurodegeneration in MJD has been described only in particular regions of the brain such as the cerebellum, substantia nigra and pontine nuclei. It has been proposed that the cellular expression of the disease gene is not in itself sufficient to cause neuronal degeneration, and that other cell-specific factors must be invoked to explain the restricted neuropathology seen in MJD [63].

#### DUBs linked to other diseases

There are also numerous examples of other DUBs linked to different diseases that have not been covered herein, owing to space constraints. These include (but are not limited to): UBP1 (encoded by *USP1*), which deubiquitylates a component of the Fanconi anemia DNA repair complex [69]; BAP1, a UCH-type DUB that binds to the BRCA1 breast-cancer susceptibility protein [70]; UBP11 (encoded by *USP11*), which binds to BRCA2 [71]; UBP4 (encoded by *USP4*), an oncoprotein linked to lung cancer [72] that interacts with the Retinoblastoma tumor suppressor proteins [73,74], and also interacts with and deubiquitylates the ubiquitin ligase RO52 [75], an autoantigen associated with the autoimmune disease Sjögren's syndrome [76]; the DUB-1 and DUB-2 family of cytokine-inducible USP-type DUBs, where DUB-2 deubiquitylates the common cytokine receptor subunit gamma(c) [77]; and the UBP2a splice variant of UBP2 (encoded by *USP2*), which regulates the stability of fatty acid synthase in prostate cancer, protects cells from apoptosis and interacts with MDM2 [78,79]. Also, while this review has focused on mammalian DUBs, there are also relevant examples from other species, such as the *Drosophila *DUB fat facets (FAF) and its substrate, the epsin ortholog liquid facets (LQF) [80,81], which play a clear role in endocytosis and Notch signaling, with implications for human disease.

## Disease, mutation, expression, knockout

Earlier biochemical assays of DUBs employed small ubiquitin (UBIQ)-ester adducts [3,4] and these were followed by gel-based or enzymatic assays based on larger UBIQ protein fusions [6]. More recently, a UBIQ-fluorescent leaving group substrate has been widely used [82,83]. UBIQ chains of defined lysine linkage have also been developed, mainly through the pioneering work of Cecile Pickart (Johns Hopkins University, Baltimore, USA) [84], and these are useful in determining DUB preference for chain topology.

Recently, RNAi-based knockdown of a large number of DUBs has been successfully employed in cell-based screens aimed at identifying roles for DUBs in regulatory pathways, such as CYLD in NFκB signaling [31] and UBP1 (encoded by *USP1*) in Fanconi anemia [69]. More recently, mice lacking specific DUBs have been developed, and these will provide useful models for further understanding the roles of DUBs in disease. For example, the CYLD- [34,35] and TNAP (A20)- [38] deficient mice provide useful models to address the role of these DUBs in inflammatory disease. Similarly, the UBP7 (*USP7*)-null mouse, which shows increased P53 (p53) levels [20], led investigators to conclude that UBP7's main substrate could be the MDM2 ubiquitin ligase, rather than P53 itself.

In two other cases, mutations in naturally occurring mouse disease models were mapped to DUB genes. The Ataxia mouse, bred for some 50 years by (and available from) the Jackson Laboratories, was found in 2002 to harbor an inactivating insertion in the proteasome-associated DUB UBP14 [51]. While it is clear that the null mutant has a neurological phenotype, it remains unclear whether this is a non-specific (proteasomal) or specific substrate effect. However, the availability of the *Usp14 *mutant mouse should accelerate discovery in this area.

Secondly, the mutation in the gracile axonal dystrophy (*gad*) mouse was mapped to a deletion in the *Uchl1 *gene [85], and subsequent work on this model revealed that UCHL1 regulates the morphology and differentiation of neural progenitor cells [86]. Thus the *Uchl1 (gad) *mouse provides a useful model for further study.

One other mouse model, the *Usp18*-null mouse, is useful in the study of interferon signaling and innate immunity against viral and bacterial infection [87]. However, it has not been covered in detail here, because UBP18 cleaves the interferon-stimulated ubiquitin-like protein UCRP (ISG15), rather than UBIQ itself.

## Disease targets and ligands

DUBs represent the newest, and least studied, family of enzymes in the ubiquitin proteasome pathway. Potentially, they have the ability to regulate the ubiquitylation status, and thus function, localization, and/or degradation, of any ubiquitylated protein, and therefore should be ideal drug targets for therapeutic intervention. However, there are no specific drugs reported for any DUB to date. Theoretically, there should be two possible routes of intervention: (i) modulation of the DUB's enzymatic activity, or (ii) modulation of the DUB's interaction with its ubiquitylated substrate and/or with the cognate ligase. There are several non-specific inhibitors of whole classes of DUBs, such as ubiquitin-aldehyde [80], which blocks at least the UCH and USP families, as well as other cysteine protease inhibitors. However, these target the catalytic protease domains of DUBs, and would at best have broad specificity. Other screens using branched peptide mimics of UBIQ-UBIQ linkages have also been performed (e.g. [88]); these may allow development of inhibitors of cleavage of specific lysine linkages. Further approaches aim to identify candidate inhibitors of deubiquitylating activity based on the X-ray crystallographic structure of DUB active sites (World Patent WO9901567). It could also be possible to stimulate DUB activity with small molecules that may enhance active site configuration, or that induce DUB gene expression, although such approaches have not been reported.

Given that the interacting regions between DUBs and their substrates (or cognate ubiquitin ligases) are now being defined, it is hoped that more rapid progress towards specific drugs will be made. For example, given the apparent primary role of UBP7 (*USP7*) in regulating MDM2 levels, and the well-defined binding sites of these proteins [21], UBP7 would be a logical choice for therapeutic intervention. Preventing UBP7-MDM2 interaction would destabilize MDM2, and thus stabilize P53 (p53) (see *DUBs and disease: UBP7/USP7/HAUSP*). This should have application in any disease where P53 is wild-type, and where stabilizing it would restore normal P53 check-point function. Such possibilities have been theoretically explored as a therapeutic approach to modulate P53 levels in hematopoietic tumors (where P53 is rarely mutated and thus amenable to manipulation [89]), but as yet, no UBP7-targeted drugs have been described. Similarly, once the full *in vivo *ramifications of the UBP20 (*USP20*)/HIF1A (HIF-1κ/VHL (pVHL) interplay are understood (see *DUBs and disease: UBP33/USP33/VDU1, UBP20/USP20/VDU2 and von Hippel-Lindau disease*), modulation of these protein-protein interactions should allow regulation of HIF1A levels in von Hippel-Lindaudisease.

## New frontiers in drug discovery

The most important issue to be addressed for most DUBs is to define the proteins that they interact with and deubiquitylate, be they (i) ubiquitylated proteins destined for degradation, trafficking, or some other consequence, or (ii) ubiquitin ligases, as it is becoming apparent that many DUBs interact with, and stabilize, ligases [19,21,44,75,79]. For the large majority of DUBs, physiological substrates remain unidentified, though it is clear from the examples discussed in this review that many DUBs have clear links to disease. This remains a major barrier to drug development. Current efforts, such as the DUB family-wide RNAi knockdown screens (e.g. [31,69]), should identify pathways that DUBs regulate, thus allowing focused efforts on substrate identification.

Once these interactions have been defined at the molecular and structural level, they can be exploited as drug targets. The P53 (p53)/MDM2/UBP7 (*USP7*) example gives a clear indication that we need to understand all aspects of a DUB's role before drugs can be rationally designed. One major unresolved question concerns the numbers of ubiquitin ligases (perhaps 500) compared with the number of DUBs (some 80–90). Does the fewer number of DUBs mean that some substrates do not have a protective DUB, and/or that DUBs are more promiscuous than ligases and can deubiquitylate several substrates, and/or that some ligases do not have a protective DUB? We need to understand the complex interplay between ubiquitin ligases, DUBs and substrates, and whether the DUB's role is to promote degradation, inhibit degradation, or is non-proteolytic, before pharmacological intervention is explored.

## List of abbreviations used

CYLD: cylindromatosis; DUB: deubiquitylating enzyme; EBNA: Epstein-Barr nuclear antigen; FOXO: forkhead box O; HAUSP: herpesvirus-associated ubiquitin-specific peptidase; JAMM: Jab1/MPN domain-associated metalloisopeptidase; MJD: Machado-Joseph disease; OUT: ovarian tumor; PD: Parkinson's disease; RIP: receptor-interacting protein; TNF: tumor necrosis factor; TRAF: tumor necrosis factor receptor-associated factor; UBP: ubiquitin-processing peptidase; USP: ubiquitin-specific peptidase; UCH: ubiquitin C-terminal hydrolase; VHL: von Hippel-Lindau.

## Competing interests

The authors declare that they have no competing interests.

## Publication history

Republished from Current BioData's Targeted Proteins database (TPdb; ).
